# Choreographing the Double Strand Break Response: Ubiquitin and SUMO Control of Nuclear Architecture

**DOI:** 10.3389/fgene.2016.00103

**Published:** 2016-06-07

**Authors:** Shane M. Harding, Roger A. Greenberg

**Affiliations:** Departments of Cancer Biology and Pathology, Abramson Family Cancer Research Institute, Basser Research Center for BRCA, Perelman School of Medicine, University of Pennsylvania, Philadelphia, PAUSA

**Keywords:** double-strand break repair, Ubiquitin, SUMO, RAP80, Telomere

## Abstract

The cellular response to DNA double strand breaks (DSBs) is a multifaceted signaling program that centers on post-translational modifications including phosphorylation, ubiquitylation and SUMOylation. In this review we discuss how ubiquitin and SUMO orchestrate the recognition of DSBs and explore how this influences chromatin organization. We discuss functional outcomes of this response including transcriptional silencing and how pre-existing chromatin states may control the DSB response and the maintenance of genomic stability.

Every organism experiences challenges to the integrity of their DNA sequence from endogenous (i.e., replication errors) and exogenous (i.e., radiation) sources. Such challenges can take the form of base mismatches and base damages or single and DSBs in the DNA backbone. Discrete molecular pathways driven by posttranslational modifications have evolved to correct each of these DNA damage types and are crucial for cellular survival and for the maintenance of genomic integrity. Perhaps the most deleterious of these lesions is the DSB as even a single unrepaired DSB can cause cell death and inaccurate repair can lead to mutations that cause cancer and other genetic diseases (Jackson and Bartek, 2009).

The DSB response encompasses multiple post-translational modifications, including ubiquitylation (ub) and SUMOylation that primarily occur within the immediate vicinity of the DSB on chromatin and chromatin-associated proteins. Locally, this promotes DSB repair mechanisms and systemically activates cellular responses, including cell cycle checkpoints that collectively suppress genomic instability. Recent technological advances and conceptual insights have highlighted how the DSB response influences the dynamic structural organization of the nucleus. In this review we will first outline how the ubiquitin and SUMOylation systems contribute to the sensing of DSBs and then examine how these pathways affect higher order chromatin structure to maintain genetic stability.

## Ubiquitin and SUMO in the DSB Response

At the apex of the molecular cascade that signals double strand breaks are three phosphatidylinositol 3-kinase related kinases (PIKKs): DNA-dependent protein kinase catalytic subunit (DNA-PKcs), Ataxia telangiectasia mutated (ATM), and ATM- and Rad3-related (ATR). DNA-PKcs forms an active holoenzyme, DNA-PK, with the heterodimer Ku70/80 at DNA ends and mainly contributes to DSB repair by non-homologous end-joining (NHEJ; Radhakrishnan et al., 2014). ATR, in cooperation with its binding partner ATRIP, binds to RPA protein-bound single stranded DNA (ssDNA) and therefore mainly senses DSBs incurred during replication where long tracks of ssDNA may be generated (Cimprich and Cortez, 2008). The most extensively characterized of this PIKK triad is ATM for which thousands of phosphorylation targets have been identified, some of which overlap with ATR and DNA-PKcs (Matsuoka et al., 2007). All three kinases are able to phosphorylate the histone variant H2A.X at serine-139 forming “γH2AX” in megabase domains surrounding DSBs. The γH2AX-laced chromatin is the platform on which the remainder of the DSB response assembles (Bonner et al., 2008).

Ubiquitin is a 76 amino acid protein that covalently modifies protein substrates through linkages between lysine residues. Ubiquitin modification is catalyzed in a pathway whereby an E1 activating enzyme passes the ubiquitin molecule to an E2 conjugating enzyme that in turn passes the ubiquitin to a substrate molecule with the help of an E3 ubiquitin ligase (Ciechanover et al., 1980; Hershko et al., 1980, 1983; Bergink and Jentsch, 2009; Popovic et al., 2014). Ubiquitin itself contains seven lysine residues that can serve as locations for chain assembly, in addition to linkages through its *N*-terminus to form linear chains (Rajalingam and Dikic, 2016). The first indications of a role for ubiquitin in the response to DNA damage came when Jentsch et al. (1987) identified a ubiquitin conjugating activity of the DNA repair gene RAD6 in *Saccharomyces cerevisiae*. Subsequently, specific ubiquitin linkages were found to be functionally relevant when a K63R mutation in ubiquitin caused sensitivity to UV and base damages of the DNA in yeast (Spence et al., 1995) and that *Y*-family DNA polymerases are recruited to UV damage through interaction with ubiquitinated PCNA (Bienko et al., 2005). A link between K63-ub chains and the DSB response in mammalian cells remained elusive until several groups identified RAP80 as a binding partner of breast cancer 1, early onset (BRCA1; Kim et al., 2007; Sobhian et al., 2007; Wang et al., 2007). RAP80 contains a tandem ubiquitin-interacting motif (UIM) that binds with high affinity to K63 linkages *in vitro* and associates with K63-linkages *in vivo* following DNA damage (Sobhian et al., 2007). Interestingly, the BRCA1-RAP80 complex is comprised of several other proteins, including MERIT40 (Feng et al., 2009; Shao et al., 2009; Wang et al., 2009) and BRCC36, a deubiquitinating enzyme with K63-ub specificity (Sobhian et al., 2007; Cooper et al., 2010; Feng et al., 2010; Patterson-Fortin et al., 2010). The structural basis for BRCC36 DUB activity has recently been solved (Zeqiraj et al., 2015) and it was also recently shown that MERIT40 deficiency is synthetic lethal in the context of BRCA2 mutation (Jiang et al., 2015). Importantly, mutations in the RAP80 UIM and in the BRCA1-RAP80 associated protein Abraxas (Nikkilä et al., 2009; Solyom et al., 2012) have been described in familial breast cancer cases where the *BRCA1* and *BRCA2* genes are not affected. These disease associated mutations highlight the importance of this specific ubiquitin interaction for genome stability.

Using siRNA-screening approaches several groups identified RING finger protein 8 (RNF8) as the first E3 to catalyse K63 linkages at DSBs in mammals (Huen et al., 2007; Kolas et al., 2007; Mailand et al., 2007; Wang and Elledge, 2007). Together these papers established that once γH2AX is generated, the mediator of DNA damage checkpoint 1 (MDC1) protein is rapidly recruited and phosphorylated by ATM at an N-terminal AQXF cluster. This phosphorylation event drives localization of RNF8 to the DSB site. Recent evidence supports a model where this rapid RNF8 recruitment drives histone H1 ubiquitylation via the UBC13 E2 ligase and this serves to recruit a second E3, RNF168, to ubiquitinate histone H2A at postions K13 and K15 (Mattiroli et al., 2012; Thorslund et al., 2015). Collectively these ubiquitylations establish chromatin changes that facilitate the recruitment of other DSB response factors including 53BP1 and BRCA1. The current models posit that in addition to providing direct docking sites for protein substrates these ubiquitin chains also drive large scale chromatin changes. One such proposition is that 53BP1 binds to pre-existing H4-K20 dimethylated residues that are exposed locally by these DSB-specific modifications (Huyen et al., 2004; Botuyan et al., 2006; Acs et al., 2011; Meerang et al., 2011; Mallette et al., 2012; Kocyłowski et al., 2015). More recent evidence demonstrates that 53BP1 is a specific reader of combinatorial histone modifications. 53BP1 DSB foci formation required H4K20 methylation by the Tudor domain as well as H2AK15-Ub recognition by a short conserved region C-terminal to the Tudor repeats called the UDR (Ubiquitin dependent recruitment) motif (Fradet-Turcotte et al., 2013). Importantly the degree of ubiquitylation surrounding DSBs is constrained in part by limited RNF168 protein levels. Deficiency in Ubr5 and TRIP12 increased RNF168 protein levels, resulting in excessive spreading of DSB ubiquitin and exhaustion of 53BP1 pools (Gudjonsson et al., 2012). It is now well established that ubiquitylation is a cornerstone of the DSB response and its precise control is essential for genome stability and tumor suppression.

In addition to ubiquitin, the small ubiquitin-like modifier (SUMO) proteins have been found to impact essentially every facet of the DNA damage response by modulating protein-protein interaction and enzymatic activity (Bergink and Jentsch, 2009). Discovered in 1996, SUMO is a small peptide that is covalently attached to proteins by E1, E2, and E3 SUMO ligases in a pathway analogous to ubiquitin conjugation (Matunis et al., 1996; Cubeñas-Potts and Matunis, 2013). The immunofluorescent and biochemical observation of SUMO1 and 2/3 isoforms at DSBs led to the identification of the PIAS1 and PIAS4 E3 SUMO ligases that drive SUMOylation of BRCA1 and 53BP1 (Galanty et al., 2009; Morris et al., 2009). Here, loss of either PIAS1 or PIAS4 severely impairs K63-ub at damage sites, reduces recruitment BRCA1 and 53BP1 and causes impaired DSB repair. Thus, in addition to ubiquitin, SUMO modifications occur at DSBs and modulate the DSB response.

Although conceptually it is easier to separate ubiquitin and SUMOylation, it is important to recognize that they can act in a combinatorial fashion. RNF4, a SUMO-targeted E3 ubiquitin ligase (STUbL), localizes to SUMO-modified MDC1 at DSB sites where its ubiquitin ligase activity is required for effective RAP80-BRCA1 recruitment as well as DSB repair and effective responses to replication stress (Galanty et al., 2012; Guzzo et al., 2012; Yin et al., 2012; Ragland et al., 2013; Gibbs-Seymour et al., 2015; Sarangi and Zhao, 2015). RAP80 itself contains a SIM domain adjacent to its UIM domains and each of these domains cooperates in the productive association of RAP80-BRCA1 to damage sites (Guzzo et al., 2012; Hu et al., 2012). Importantly, BRCA1 itself is an E3 ubiquitin ligase that can catalyze K6 linkages of ubiquitin *in vitro* and this activity is stimulated by SUMOylation of BRCA1 (Wu-Baer et al., 2003; Morris and Solomon, 2004; Polanowska et al., 2006; Morris et al., 2009). Although the function of this particular BRCA1 activity is not well understood this serves as a clear example of the interconnection of ubiquitin and SUMOylation in the DSB response (Messick and Greenberg, 2009; Jackson and Durocher, 2013).

Recent proteomic studies have identified hundreds of damage-induced targets of both ubiquitylation and SUMOylation (Psakhye and Jentsch, 2012; Elia et al., 2015). The array of targets and the potential for combinatorial effects of these moieties poses a challenge to understanding how a particular modification on a particular protein impacts the DSB response. This problem may be particularly true for SUMOylation as one of these reports suggests that it is the bulk SUMOylation of a group of proteins rather than any one specific target that stimulates DSB repair (Psakhye and Jentsch, 2012). Despite these challenges it is immediately apparent that phosphorylation, ubiquitylation, and SUMOylation make the chromatin permissive to recruit the various effectors of the DSB response that collectively activate repair mechanisms and cell cycle checkpoints. More recently several groups have begun to explore how the DSB response impacts higher order chromatin structure and nuclear architecture that are strongly influenced by these and other post-translational modifications.

## DSB Response-Driven Nuclear Reorganization

**FIGURE 1:**
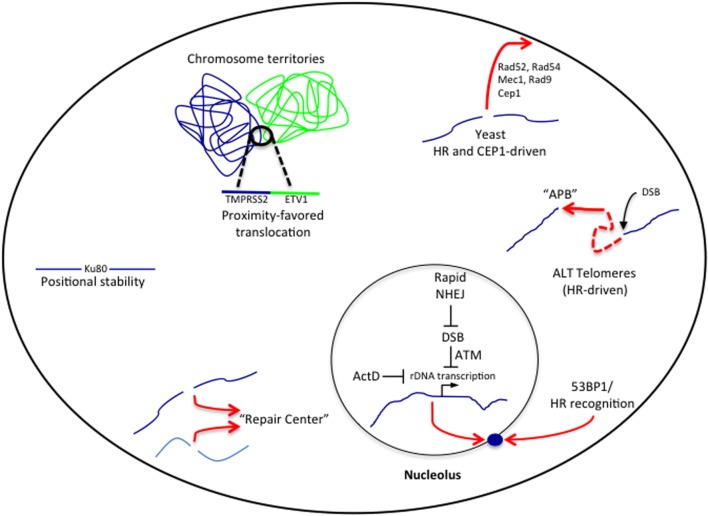
**Chromatin reorganization during double strand break (DSB) responses.** In several contexts, DSBs induce chromatin reorganization. Errors in DSB repair can result in translocations that occur most frequently between chromosomes that are already in close spatial proximity through pre-existing organization into chromosome territories. Moving clockwise, persistent breaks in yeast are relocalized to the nuclear periphery in a SUMO dependent process for repair by homologous recombination (HR). Homology directed repair driven movement of DSBs induced at telomeres promotes clustering into ALT Promyelocytic Bodies (APBs). In nucleoli persistent breaks silence transcription, which leads to relocalization of the rDNA to the nucleolar periphery. In some instances multiple breaks appear to localize to “repair centers” while in other contexts breaks remain positionally stable.

For the purposes of this review, we will separate chromatin reorganization into two interconnected categories. The first category includes large-scale chromatin redistributions in which the damaged locus changes its physical location within the 3D nuclear compartment (**Figure 1**). For example, moving from the nuclear interior to the periphery. We will discuss how these particular movements may influence the mechanism and fidelity of double strand break repair. These movements have been summarized in **Table 1**. The second category includes more localized epigenetic changes that cause transition between heterochromatic and euchromatic states. In this instance we will focus our discussion on recent insights into how these changes influence transcription near the DSBs.

**Table 1:** Large-scale movement following double strand breaks (DSBs).

DSB location/break method	Movement	References
**Yeast**		
rDNA	To nucleolar periphery	Torres-Rosell et al., 2007
MAT Locus	To nuclear periphery	Kalocsay et al., 2009
MAT Locus	To nuclear periphery	Nagai et al., 2008
Single I-SceI site	Increased local mobility	Dion et al., 2012; Neumann et al., 2012
MAT Locus	Increased local mobility	Strecker et al., 2016
**Mammalian**		
α-radiation	Break clustering	Aten et al., 2004; Stap et al., 2008
γ-radiation	Break clustering	Neumaier et al., 2012
γ-radiation, etoposide	Local mobility	Krawczyk et al., 2012
Charged nuclei, nuclease	Local mobility	Becker et al., 2014; Caron et al., 2015
UV-microbeam, γ-radiation	Chromatin decondensation	Kruhlak et al., 2006; Falk et al., 2007
Charged nuclei	Minimal	Jakob et al., 2009
Ultrasoft *X*-rays	Minimal	Nelms et al., 1998
Single multicopy locus	Minimal	Soutoglou et al., 2007
I-SceI Chr1, 7, 10	Loci pairing	Roukos et al., 2013
I-PpoI	Homolog pairing	Gandhi et al., 2012
I-PpoI rDNA	To nucleolar periphery	Harding et al., 2015; van Sluis and McStay, 2015; Warmerdam et al., 2016
		
Telomere deprotection	Telomere fusion	Dimitrova et al., 2008; Lottersberger et al., 2015
TRF-FokI ALT Telomeres	Telomere clustering	Cho et al., 2014

### Chromatin Movement and the DSB Response: Gross Chromatin Movements

Textbook descriptions of chromatin are, by necessity, static depictions of linear DNA bound by histones and other factors. Even in undamaged DNA this static arrangement is inaccurate and several mathematical models based on cellular data have described active chromatin movement as a non-directional random walk over relatively short distances (Dion and Gasser, 2013). These short-range movements are constrained by multiple cellular and physical properties and lead to occupancy of chromosomes within non-randomly defined nuclear volumes called “chromatin territories” (Cremer and Cremer, 2010). Less frequent longer-range movements have also been detected in various contexts. For example, targeting of the VP16 transcriptional activator to the nuclear periphery resulted in movement to the nuclear interior and inhibition of RNA Polymerase I (Pol I) transcription causes relocalization of chromatin to the nucleolar periphery (Tumbar and Belmont, 2001; Floutsakou et al., 2013).

In yeast, there is clear evidence that DSBs induce chromatin mobility. When DSBs were induced in the rDNA of *S. cerevisiae* these breaks relocalized to the exterior of the rDNA-containing nucleolus (Torres-Rosell et al., 2007). As even undamaged rDNA repeats transiently moved outside of the nucleolus the authors proposed that it was this underlying dynamic motion rather than a specific DSB-driven process led to the translocation. Importantly, this movement was dependent on a specific SUMOylation event in RAD52 that is also required for homology-directed repair (HR) of these rDNA loci. At other genomic loci in yeast SUMOylation also targets DSBs at defined genomic sites to the nuclear periphery (Nagai et al., 2008; Kalocsay et al., 2009). Breaks elsewhere in the yeast genome also led to a greater mobility of chromatin that was dependent on RAD51, RAD54, MEC1, RAD9 (similar to human MDC1, 53BP1 and BRCA1), and INO80 (Dion et al., 2012; Neumann et al., 2012). A recent report also found that the INO80-driven movement of DSBs within subtelomeres depends on actin polymerization (Spichal et al., 2016). These movements at least in part contributes to homology searches during HR (Miné-Hattab and Rothstein, 2012). A recent study found that DSB-induced MEC1-driven phosphorylation of the kinetochore component Cep1 causes release of the centromere from the spindle pole body and facilitates chromatin movement (Strecker et al., 2016). Additionally, the authors found that tethering of telomeres to the nuclear periphery constrains chromatin movement and the physical breakage of the chromatin from this linkage facilitates further chromatin mobility. Interestingly, in this instance the authors found no evidence for an HR defect but rather propose that the increased mobility facilitates cell cycle checkpoint activation. Therefore, a preponderance of evidence exists that DSBs in yeast are mobile and that SUMOylation and the DSB response drive this mobility. Even if the precise functional outcome of this movement is unclear, the consensus is that the movement has a positive impact on the ability of the yeast cell to survive DSBs and therefore sets precedent for study in mammalian cells.

There are now numerous reports of chromatin mobility in response to DSBs in mammalian cells, albeit the determinants for this mobility are incompletely defined, as many breaks appear to be stable in their nuclear position. One of the first examples of subnuclear DSBs induced by soft *X*-rays suggested that breaks are positionally stable during the initial phases of the damage response although the temporal and spatial resolution with this method is limited (Nelms et al., 1998). Using α-radiation to create DSBs along a confined linear track of the nucleus, Aten et al. (2004) found that breaks redistributed into clusters giving rise to MRE11 dependent “repair centers” that were most predominant in G1-phase (Stap et al., 2008). Similarly, DSBs created by γ-rays or etoposide induced movement of damaged chromatin >2 fold over that of undamaged loci (Krawczyk et al., 2012). Evidence for repair centers have also been reported in which IR induced GFP-53BP1 foci between 1 and 2 μm apart can rapidly gather into larger clusters (Neumaier et al., 2012). Loss of ATM reduced movements at both γ-rays and by charged nuclei (Becker et al., 2014) and at nuclease induced breaks (Caron et al., 2015). Conversely, DSBs induced by UV-microbeam or γ-rays were found to have limited mobility but led to a localized decondensation of chromatin (Kruhlak et al., 2006; Falk et al., 2007). Induction of multiply damaged sites (containing DSBs, single strand breaks and base damages) by charged nuclei was not found to cause significant movements nor did DSBs induced by a nuclease at an engineered multicopy transgene locus of likely >100 repeats that is heterochromatic (Soutoglou et al., 2007; Jakob et al., 2009). The reason for such discrepancy is not clear but may be related to cell types, modes of damaged induction employed, imaging methods used or the method to visualize the DSBs themselves. One plausible explanation is that the loci broken in the nuclease experiments were repetitive transgenes that did not share homology with sequences on different chromosomes. In this scenario, homology directed mobility and clustering would not be possible. Interestingly, the constraint on mobility was dependent on Ku80, a component of NHEJ repair of DSBs (Soutoglou et al., 2007). This suggests that the NHEJ machinery tethers or rapidly rejoins DSB ends to limit mobility; this may also underlie movements when breaks are induced in the nucleolus as discussed below. However, an additional report using such transgenes described long distance MRE11 dependent mobility that was associated with chromosome translocations (Roukos et al., 2013), perhaps related to the original reports from Aten et al. (2004). Interestingly, I-PpoI nuclease induced breaks were found to cause pairing of homologous genetic loci in an ATM and transcription dependent manner in G1 phase of the cell cycle (Gandhi et al., 2012). Although canonical HR dependency was not examined, a possible explanation is the occurrence of homology directed clustering in G1 due to the absence of a sister chromatid. It is also important to note that the intercomparison of studies is difficult due to differences in measurement methods and the lack of standard comparators for movement in undamaged chromatin. The differences observed between yeast and mammalian cells may, in part result from the balance in repair pathways used. Yeast preferentially use HR, the less error prone mechanism of DSB repair that uses a sister chromatid as a template to resolve the break. NHEJ, the more error prone pathway that relies on the direct rejoining of broken ends, is more predominant in mammalian cells (Shrivastav et al., 2008). These differences in repair pathway between species may have important implications for the outcome of DSB responses in mammalian cells, as described below.

In light of these issues, one clear context where DSB movement occurs is at deprotected and damaged telomeres. To prevent their recognition as DSBs telomeres are protected in a complex called shelterin that blocks access to the ends by the DSB machinery (Palm and de Lange, 2008). When shelterin is depleted the DSB response is activated and telomeres are joined by NHEJ to cause striking telomere fusions (Doksani and de Lange, 2014). Loss of 53BP1 reduced the mobility of these telomere ends and resulted in almost complete loss of telomeric fusions (Dimitrova et al., 2008). These movements are driven at least in part by the LINC complex which connects dynamic microtubules to the inside of the nucleus; similar movements were also described at non-telomeric DSBs generated in BRCA1 deficient cells using an inhibitor of Poly (ADP-ribose) polymerase (PARPi) and this was proposed to contribute to 53BP1-dependent interchromosomal NHEJ (Lottersberger et al., 2015).

A second striking example of DSB dependent chromatin mobility also occurs at telomeres, but in a 53BP1 independent manner. Approximately 10–15% of cancer cells employ “alternative lengthening of telomeres (ALT)” to maintain their telomere length. Rather than activating telomerase, ALT cells utilize a homology driven mechanism to promote lengthening of telomeres (O’Sullivan and Almouzni, 2014; Dilley and Greenberg, 2015; Pickett and Reddel, 2015). Our laboratory recently developed a method whereby DSBs are generated specifically in telomeres to elicit a DSB response (Tang et al., 2013). In ALT, but not telomerase positive cells, such DSBs induced directed movement of telomere ends into clusters called ALT-associated PML bodies (APBs), a hallmark of ALT cells (Cho et al., 2014). Unlike other DSB movements described to date the movement in this case was biphasic. During the first phase damaged telomeres showed a significant increase in mobility as compared to undamaged telomeres; this mobility is similar to those movements described above. In the second phase the “incoming” telomere makes a long-range directed movement toward a relatively immobile “recipient” telomere. Both phases of this movement were dependent in part on the HR machinery (e.g., RAD51) and also on Mnd1-Hop2, a complex generally involved in meiotic interhomolog recombination. In agreement with these findings, ALT telomere replication stress due to SMARCAL1 deficiency also resulted in Rad51 telomere–telomere clustering and dramatic telomere enlargement (Cox et al., 2016). These results highlight the first example of directed DSB movement in mammalian cells mediated by HR, and collectively, reveal that dynamic chromatin movements contribute to genomic stability and cellular immortality through telomere maintenance.

In several contexts, it is clear that DSBs can induce chromatin movement. How the DSB response itself impacts these movements is slowly beginning to be elucidated. Unlike SUMOylation, a direct role for ubiquitin in DSB movement has not been described. The involvement of MEC1 and RAD9 in yeast implies that this may be the case. Although RAD52 is a clear SUMOylation target in yeast, it is also possible that movements are controlled by other SUMOylation events or simply by bulk SUMOylation of multiple factors (Psakhye and Jentsch, 2012). In *Drosophila melanogaster*, heterochromatic breaks are mobilized from heterochromatin to the nuclear periphery in a mechanism mediated by the STUbLs Dgrn and Rad60 (Chiolo et al., 2011; Ryu et al., 2015). This is reminiscent of the SUMO-dependent relocalization of yeast DSBs to the periphery of the nucleus or nucleolus suggesting there is conservation of the mechanism across species. As in yeast we expect that in addition to recruiting DSB response factors for cell signaling and DSB repair, ubiquitylation and SUMOylation events serve to modulate chromatin movement. Indeed, at ALT telomeres the MMS21 subunit of the SMC5/6 complex SUMOylates multiple telomere binding proteins (e.g., TRF1) and contributes to HR of ALT telomeres and localization to APBs (Potts and Yu, 2007). It is also possible that the control of DSB movement is a fundamental aspect of the DSB response that controls the homology search during HR (as in telomeres described above) and may also limit illegitimate NHEJ as described below.

### Chromatin Movement and the DSB Response: Localized Responses

The development of chromosome conformation capture (3C) and related high-throughput “C” technologies (e.g., Hi-C) has allowed the refinement of the chromosome territory models described above. These methods allow the interrogation of chromosome contacts that occur both within and between chromosomes. The most obvious of these contacts occur within topological associated domains (TADs) that are intrachromosomal regions of hundreds of kilobases in mammals that contain within them genes with similar expression dynamics (Dekker and Misteli, 2015). These TADs appear to arrange chromatin into regions whereby long-range interactions, such as between enhancers and promoters, can occur. Although markedly less frequent than TADs, interchromosomal contacts occur most often between chromosomes that are within the same chromosome territories as defined by FISH chromosome painting (Lieberman-Aiden et al., 2009). These concepts paint a picture of the nucleus whereby hierarchical levels of organization arrange chromatin in a dynamic non-random fashion. More recently indications that this organization can influence the DSB response itself and the outcome of DSB repair have arisen with implications for genetic stability and the generation of genetic abnormalities associated with cancer.

Studies of local chromatin dynamics at breaks are in their infancy. In yeast, 3C studies suggest that DSBs reduce the overall frequency of local (<100kb) interactions (Oza et al., 2009). This decrease appears to be correlated with the HR-dependent movement of the breaks to the nuclear periphery, as in G1-arrested cells where HR is inactive the interaction frequencies were less dynamic. This led to the proposal that damaged DNA is sequestered from the local chromatin environment to facilitate accurate DSB repair. This model is consistent with recent findings in mouse B-cells. By arresting cells in G1 to eliminate confounding HR-driven repair mechanisms, DSBs within a given chromosome most frequently led to translocation with genomic loci present *in cis* to the breaks (Hakim et al., 2012; Zhang et al., 2012).

A practical example of how differential localization can influence genome stability occurs in prostate cancer cells. When stimulated with dihydrotestosterone (DHT) TMPRSS2 gene expression is strongly induced in a manner dependent on topoisomerase II (TOP2) catalyzed DSBs that relieve torsional stresses that block transcription (Gómez-Herreros et al., 2014). These TOP2 dependent breaks have recently been mapped and frequently occur at breakpoints that are present in clinical fusions of TMPRSS2 with ETS transcription factors (e.g., ERG; Haffner et al., 2010). Linking back to nuclear organization, the TMPRSS2 and ETS transcription factor loci are frequently associated within the nuclear space (Lin et al., 2009; Mani et al., 2009). Consistent with the proximity model, fusions of TMPRSS2 to ERG (both localized on chromosome 21) occur in ~90% of fusion cases whereas fusion to ETV1 (located on Chromosome 7) occurs at a much lower frequency (Tomlins et al., 2007). Thus chromosomal proximity can underlie translocations that are characteristic of cancer-associated genomic instability. Interestingly, mutations in TDP2, the enzyme that removes TOP2 that becomes trapped on DNA ends, results in persistent breaks and a human syndrome (Gómez-Herreros et al., 2014). Etoposide (a drug that traps TOP2 on broken DNA ends) causes DSBs that require TDP2 for break repair and resumption of transcription at TOP2 dependent loci, such as TMPRSS2. Indeed, TDP2 deficient mice also showed defective recovery of transcription in the developing mouse brain that was correlated with a reduction in the density of interneurons of the cerebellum. Given the clear relationship between transcription, a known modulator of local chromatin structure, and the DSB response it will be of prime interest to understand how these two interrelated cellular events impact on the higher order chromatin structure in combination and how this influences carcinogenesis and neurodevelopment.

It is becoming clear that the dynamic organization in 3D space of the nucleus has a direct influence on genomic stability and the DSB response. As technologies advance and methods for localized induction of DSBs in mammalian cells mature it will become possible to examine how these local chromatin interactions influence, and are influenced by, the DSB response.

## Communication between the DSB Response and Transcription

Post-translational modifications on histones are a cornerstone of the DSB response. This has stimulated considerable interest in epigenetic marks on chromatin in the vicinity of the break site and the functional outcomes that this can entail. Recent studies from several labs have identified specific histone modifications driven by the DSB response that modulate transcription near the DSB site. We will outline some of these histone modifications in different physiological contexts and briefly discuss the functional outcomes of these events.

During meiosis SPO11, a TOP2-like enzyme, creates multiple DSBs to drive pairing between homologous chromosomes that initiates HR to induce crossovers and genetic variation (Lu and Yu, 2015). In males the *X*- and *Y*-chromosomes lack partners and remain largely unpaired during meiosis but remain replete with DSBs that activate a DSB response in an isolated structure called the *XY-*body (Turner, 2007). These DSBs are resected to initiate recombination events and are substrates for ATR activation and γH2AX formation throughout the sex chromosomes (Turner et al., 2004). As in mitotic cells this γH2AX laced chromatin recruits MDC1 and RNF8, although whether RNF8 localization is strictly dependent on MDC1 in this context is unclear (Ichijima et al., 2011). In the *XY*-body MDC1 contributes to amplification of γH2AX signals and also induces SUMOylation but the target(s) remain undefined (Ichijima et al., 2011). As in somatic cells RNF8 drives H2A-ub formation in the *XY*-body and 53BP1 recruitment, however, BRCA1 does not spread throughout the *XY*-body but rather amplifies upstream signaling of ATR to γH2AX (Turner et al., 2004; Sin et al., 2012).

A striking outcome of this meiotic DSB signaling is the transcriptional silencing of genes on the *X*- and *Y*-chromosomes, a processed called meiotic sex chromosome inactivation (MSCI). MSCI occurs at least in part due to histone modifications including H2A-ub, H3 and H4 deacetylation, and H3K9 dimethylation; these marks persist during silencing throughout meiosis even after γH2AX has been resolved (Turner, 2007). MSCI fails in MDC1, H2AX, and BRCA1 null mice which correlates with male infertility, highlighting the role of transcriptional silencing in this context (Fernandez-Capetillo et al., 2003; Turner et al., 2004; Ichijima et al., 2011). Interestingly, RNF8 mice appear to maintain MSCI, however, males display reduced fertility possibly owing to other aspects of RNF8 dependent signaling in spermatogenesis (Li et al., 2010; Lu et al., 2010). These data highlight the importance of the DSB response in silencing transcription during meiosis to facilitate productive spermatogenesis.

The first study to suggest crosstalk between DSBs and transcription in somatic mammalian cells observed decreased RNA Pol I transcription in nucleoli of irradiated cells (Kruhlak et al., 2007). This silencing was dependent on ATM, MDC1, and NBS1 and prolonged in repair-deficient cells. Silencing in this context was independent of H2AX but the reasons for this uncoupling of the H2AX-MDC1 axis are unclear.

Persistent DSBs also silence transcription from RNA PolII-dependent promoters. Our group developed a system to simultaneously visualize DSB responses and nascent transcription in U2OS cells. Multiple breaks are induced within a LacO cassette 4 kb upstream of an inducible transcriptional unit in which the 3′-UTR (untranslated region) harbors 24 repeats of a stem loop that is recognized by the phage coat protein MS2 (Janicki et al., 2004; Shanbhag et al., 2010). This enables real time visualization of the DSB site and nascent transcription through the expression of mCherry-LacIFokI and YPF-MS2 fusion proteins, respectively. Introduction of a LacI molecule fused to the FOKI endonuclease creates a robust DSB response upstream of the transcriptional start site that effectively silences RNA PolII dependent transcription in an ATM and ubiquitin dependent manner. PolII was maintained at the locus, however, showed reduced levels of phosphorylation at the Serine 2 position of its carboxy terminal domain repeats, indicating impaired transitioning to elongating forms. This effect was strongly dependent on ATM and associated with H2A ubiquitylation. Transcription was rapidly restored upon nuclease termination and DSB repair, but persisted in the absence of the H2A-ub specific DUB USP16. Interestingly, deficiency in either RNF8 or RNF168 did not impact DSB silencing, albeit a modest reduction in silencing occurred upon combined knockdown. This implied that although these specific ubiquitylation events contribute to DSB silencing, other ATM-dependent events likely cooperate in suppressing transcription. This suggestion was recently supported by the finding that ATM-dependent phosphorylation of BAF180, a component of the chromatin remodeling PBAF complex, was required for H2AK119-ub and transcriptional silencing (Kakarougkas et al., 2014). Furthermore, depletion of BMI1 and EZH2, components of polycomb repressive complexes (PRC) 1 and 2, respectively, also contributed to DSB silencing (Ui et al., 2015). Importantly, ATM-dependent phosphorylation of ENL enhanced its interaction with BMI1 (i.e., PRC1) and led to transcriptional silencing. Together these data produce a model whereby multiple ATM-dependent signaling events lead to chromatin modifications that silence transcription *in cis* to DSBs. Interestingly, recent reports showed that transcriptional silencing of rDNA can occur *in trans* when DSBs are induced by UV-microbeam or IR (Ciccia et al., 2014; Larsen et al., 2014). Other studies have identified site-specific small RNAs generated by DICER-DROSHA in mammalian cells and in *Arabidopsis thaliana* that facilitates recognition of DSBs or repair by HR (Francia et al., 2012; Wei et al., 2012; Gao et al., 2014). It is important to note that this production of small RNAs at DSBs is distinct from silencing of RNA-PolI and PolII driven genes, as the ncRNAs do not appear to be promoter driven. Future studies exploring how specific DSBs influence transcriptional silencing both *in cis* and *in trans* on a global scale will be required to fully understand the extent of DSB silencing of promoter-driven transcription and in the production of small RNAs derived from the local chromatin.

During studies with our transcriptional reporter system we found that ATM-dependent silencing suppressed transcriptionally induced chromatin decondensation (Shanbhag et al., 2010). Despite observing overall positional stability of DSBs, Kruhlak et al. (2006) observed local expansion of chromatin following both IR and UV-microbeam damage. This correlated with decreased DNA density in electron microscopy but was independent of ATM and H2AX. In another example *D. melanogaster* IR induced local decondensation of HP1a-associated heterochromatin and this was proposed to facilitate DSB repair (Chiolo et al., 2011). At first, the finding that the DSB response can suppress transcription associated chromatin decompaction and that DSBs themselves induce decompaction appear at odds. However, it is highly likely that the pre-existing state of chromatin at the time of DSB induction influences the nature of the DSB response and the outcome of ATM signaling. To reconcile these issues it will be important to develop systems whereby DSBs can be induced within different chromatin states in the same biological system to determine how this influences chromatin dynamics.

## Chromatin Reorganization as a Requirement for DSB Repair

DSBs are primarily repaired by one of two pathways in mammalian cells. In late S- and G2-phases after replication has taken place, there is competition between rapid NHEJ and slower but more accurate HR. Understanding this balance has long been a goal of studies in the DSB repair field.

Tightly packed heterochromatin structures have been thought to be barriers to DSB repair and radioresistance (Chapman et al., 1999; Schaue and McBride, 2015). One possible reason for this is the limited accessibility of repair factors to highly compact chromatin structures. Indeed, it has often been observed that γH2AX preferentially forms in less dense euchromatin (Cowell et al., 2007). As mentioned above, correlative light and electron microscopy have demonstrated chromatin decompaction and that nucleosomes are disrupted in the vicinity of DSBs (Goldstein et al., 2013). Recently a pathway dependent on ATM that mediates DSB repair in heterochromatin regions has been described. It had long been known that ATM null cells repair the majority of DSBs (~85%) with normal kinetics but that the remaining ~15% of breaks remain unrepaired for long times after damage (Riballo et al., 2004). When analyzed by immunofluorescence of γH2AX in mouse cells these residual DSBs localize adjacent to heterochromatic “chromocenters” and required ATM-dependent phosphorylation of the KAP1 protein for resolution (Goodarzi et al., 2008). KAP1 is a component of heterochromatin and its phosphorylation by ATM drives relaxation of heterochromatin (Ziv et al., 2006). Phosphorylated KAP1 is maintained by the RNF8-RNF168-MDC1-53BP1 pathway and thus links ubiquitylation to DSB repair in heterochromatin.

As mentioned, translocations driven by NHEJ occur largely between chromosomes in close spatial proximity (Hakim et al., 2012; Zhang et al., 2012; Roukos et al., 2013). To prevent this happening at high frequency one may predict that the localization of chromatin on multiple levels must be controlled. In yeast, where HR is the dominant mechanism of repair, movement is viewed as a priority for DSB repair (Miné-Hattab and Rothstein, 2013). In mammals, where NHEJ predominates, limitations on movement may be necessary to prevent unwanted rejoining, but movement is observed in certain circumstances. As an example, breaks induced at the nuclear membrane were found to be positionally stable and did not relocalize to environments that were more permissive for HR rather they were repaired by alternative end-joining in place (Lemaître et al., 2014). Recently, studies from two laboratories generated DSBs within nucleoli of mammalian cells using endonucleases (Harding et al., 2015; van Sluis and McStay, 2015). In each case these DSBs and the rDNA chromatin itself were detected at the periphery of nucleoli indicating movement had occurred. This movement was associated with transcriptional silencing and when this silencing was blocked by inhibition of ATM the reorganization of nucleoli and the rDNA was prevented (**Figure 1**). We found that when NHEJ was blocked nucleolar reorganization and transcriptional silencing was enhanced; this was not observed when HR was inhibited. This suggested that NHEJ was the predominant mode of DSB repair in nucleoli, which was borne out by direct repair assays at the rDNA loci. Interestingly van Sluis and McStay (2015) observed HR-associated replication at the nucleolar periphery suggesting a role for HR in rDNA repair. Inefficient repair of rDNA by HR was also found to generate a loss of rDNA repeats; this effect was exacerbated by loss of NHEJ (Warmerdam et al., 2016). These complementary studies suggest that NHEJ occurs rapidly within nucleoli to maintain rDNA transcription. However, when these breaks remain unrepaired by NHEJ they are transcriptionally silencing and relocalize to the nucleolar periphery where they can be recognized by the HR machinery in a deleterious repair mechanism. Thus, DSBs in the rDNA recapitulated to some extent breaks in yeast where redistribution facilitates HR. This serves to highlight the role of nuclear organization in regulation of DSB repair pathway choice and may be a useful model system in which to study how ubiquitin and SUMO contribute to repair by NHEJ within the nucleolus and HR in the nucleolar periphery.

## Perspectives

Over the last 30 years the mechanisms of the DSB response have been intensively studied and have provided an intricate model for the recognition and subsequent repair of DSBs dependent on post-translational modifications including phosphorylation, ubiquitylation, and SUMOylation. Of particular interest in the coming years will be how each of these modifications act in combination to drive accurate recognition of the breaks and repair pathway choice. SUMOylation and ubiquitylation offer a prime example of such concerted actions that are just beginning to be understood. Although technically challenging understanding how these multifaceted interactions are orchestrated is key to fully elucidating the DSB response. Recent evidence from many groups has begun to unravel these issues, and invariably they require multiple modifications rather than a single chromatin mark highlighting the importance of viewing the DSB response holistically rather than as singular distinct pathways.

Technological advances in the last decade have provided the tools necessary to interrogate how the organization of the nucleus both at the global (i.e., chromosome interaction) level and at the level of the epigenome. Key consideration in this regard include how the DSB response modulates chromatin interactions during the acute phase of the DNA damage response, and if persistent DNA damage signaling alters the epigenome. Equally important will be understanding how dynamic movements in the mammalian nucleus are controlled following DNA damage. Given recent evidence that such movements are important for the generation of chromosomal translocations a molecular understanding, such as that emerging in yeast, will be a fruitful area of future study.

It has also become increasingly apparent that the context (nuclear location, chromatin states, etc.) in which a DSB is induced has a significant effect on the nature of the response and outcome of repair. Several experimental approaches are now available to induce breaks within defined chromatin environments and physical locations. These systems will undoubtedly facilitate a broader understanding of the contextual aspects of the DSB response and will lead to a more unified model of nuclear organization, cell signaling, and DSB repair.

## Author Contributions

All authors listed, have made substantial, direct and intellectual contribution to the work, and approved it for publication.

## Conflict of Interest Statement

The authors declare that the research was conducted in the absence of any commercial or financial relationships that could be construed as a potential conflict of interest.
